# Evaluating fatigue in patients recovering from COVID-19: validation of the fatigue severity scale and single item screening questions

**DOI:** 10.1186/s12955-022-02082-x

**Published:** 2022-12-27

**Authors:** Hiten Naik, Selena Shao, Karen C. Tran, Alyson W. Wong, James A. Russell, Esther Khor, Luis Nacul, R. Jane McKay, Christopher Carlsten, Christopher J. Ryerson, Adeera Levin

**Affiliations:** 1 Department of Medicine, University of British Columbia, 2775 Laurel Street, Vancouver, BC V5Z 1M9 Canada; 2 Provincial Health Services Authority, Vancouver, Canada; 3 Division of General Internal Medicine, University of British Columbia, Vancouver, Canada; 4 Centre for Heart Lung Innovation, St. Paul’s Hospital, University of British Columbia, Vancouver, Canada; 5 Division of Critical Care Medicine, University of British Columbia, Vancouver, Canada; 6 Vancouver Coastal Health Research Institute, Vancouver, Canada; 7 Division of Nephrology, University of British Columbia, Vancouver, Canada

**Keywords:** Fatigue severity scale, Long COVID, SARS-CoV-2, Quality of life, Psychometrics

## Abstract

**Background:**

Fatigue is a common symptom in hospitalized and non-hospitalized patients recovering from COVID-19, but no fatigue measurement scales or questions have been validated in these populations. The objective of this study was to perform validity assessments of the fatigue severity scale (FSS) and two single-item screening questions (SISQs) for fatigue in patients recovering from COVID-19.

**Methods:**

We examined patients ≥ 28 days after their first SARS-CoV-2 infection who were hospitalized for their acute illness, as well as non-hospitalized patients referred for persistent symptoms. Patients completed questionnaires through 1 of 4 Post COVID-19 Recovery Clinics in British Columbia, Canada. Construct validity was assessed by comparing FSS scores to quality of life and depression measures. Two SISQs were evaluated based on the ability to classify fatigue (FSS score ≥ 4).

**Results:**

Questionnaires were returned in 548 hospitalized and 546 non-hospitalized patients, with scores computable in 96.4% and 98.2% of patients respectively. Cronbach’s alpha was 0.96 in both groups. The mean ± SD FSS score was 4.4 ± 1.8 in the hospitalized and 5.2 ± 1.6 in the non-hospitalized group, with 62.5% hospitalized and 78.9% non-hospitalized patients classified as fatigued. Ceiling effects were 7.6% in the hospitalized and 16.1% in non-hospitalized patients. FSS scores negatively correlated with EQ-5D scores in both groups (Spearman’s rho − 0.6 in both hospitalized and non-hospitalized; *p* < 0.001) and were higher among patients with a positive PHQ-2 depression screen (5.4 vs. 4.0 in hospitalized and 5.9 vs. 4.9 in non-hospitalized; *p* < 0.001). An SISQ asking whether there was “fatigue present” had a sensitivity of 70.6% in hospitalized and 83.2% in non-hospitalized patients; the “always feeling tired” SISQ, had a sensitivity of 70.5% and 89.6% respectively.

**Conclusions:**

Fatigue was common and severe in patients referred for post COVID-19 assessment. Overall, the FSS is suitable for measuring fatigue in these patients, as there was excellent data quality, strong internal consistency, and construct validity. However, ceiling effects may be a limitation in the non-hospitalized group. SISQs had good sensitivity for identifying clinically relevant fatigue in non-hospitalized patients but only moderate sensitivity in the hospitalized group, indicating that there were more false negatives.

**Supplementary Information:**

The online version contains supplementary material available at 10.1186/s12955-022-02082-x.

## Background

It has been increasingly recognized that a significant proportion of patients experience persistent symptoms months after being infected with the virus that causes COVID-19, regardless of whether they were hospitalized for their initial illness [1]. In many studies, fatigue is the most reported symptom [1, 2, 3, 4]. However, reports regarding the proportion of patients that endorse fatigue has varied greatly, from as low as 1.8% in one study to as high as 98% in another [1, 2, 3, 4].

This variability could be explained in part by differences in the study populations and sampling biases, but also the means in which fatigue has been assessed [2]. In many of the observational studies thus far, fatigue was evaluated via interview or questionnaire as a single item as part of a symptom inventory [4, 5, 6, 7, 8, 9, 10]. For example, in a highly-cited internet-based survey, respondents were asked to indicate the presence or absence of fatigue among a list of over 200 symptoms [7] Other studies have used several different standardized instruments including the fatigue severity scale (FSS) [11, 12, 13, 14] chandler fatigue scale (CFQ) [15, 16], modified fatigue impact scale (MFIS) [17, 18] and the patient-reported outcomes measurement information system (PROMIS) global health instrument [19]. The heterogeneity of fatigue measurement in the literature has made it difficult to perform cross-study comparisons regarding the prevalence of fatigue and appreciate the severity of the fatigue reported [2]. It is also unclear how well the single item screening questions (SISQs) used in the symptom inventories can identify fatigue in relation to the more detailed instruments.

To better characterize post-COVID-19 fatigue and assess the efficacy of interventions, further research studies worldwide would benefit from using survey instruments that have been specifically validated in this population. Although several fatigue scales exist, none have been validated in either a previously hospitalized or non-hospitalized post-COVID-19 cohort.

Our current study focused on validation of the fatigue severity scale (FSS); a self-reported questionnaire designed to assess fatigue severity based on its impact on a patient’s functioning [20]. The FSS is one of the most used measures of fatigue and has been validated in several health conditions [21]. An evaluation of the psychometric properties of the FSS specifically in post-COVID-19 patients would help provide researchers with an understanding of the strengths and weaknesses of the instrument when designing future studies and interpreting their results.

We sought to use data collected through the Post COVID-19 Recovery Clinics (PCRCs) in British Columbia (BC) to investigate the performance of the FSS in patients that were hospitalized for COVID-19 and in patients who were not hospitalized, but referred for persistent symptoms. As such, this cross-sectional study had two main objectives. The first aim was to assess the psychometric properties of the FSS in these patient groups, including data quality, internal consistency, and construct validity. The second aim was to determine how effective two different SISQs were at identifying fatigued patients using an FSS cut-off as the reference standard.

## Methods

### Participants and data collection

The study was conducted through the Post-COVID-19 Interdisciplinary Clinical Care Network (PC-ICCN) which was designed as a learning health system to facilitate both clinical care and research throughout BC, a Canadian province of approximately 5 million people [22, 23]. At the time of this study, the PC-ICCN comprised of 4 PCRCs which were physically located in the outpatient departments at St. Paul’s Hospital, Vancouver General Hospital, Surrey Memorial Hospital and Abbotsford Regional Hospital. At the clinics, patients are assessed by internal medicine physicians in-person or by telehealth.

Patients were eligible to be referred by a clinician to the PC-ICCN if they were adults, and were either hospitalized for acute COVID-19 or were not hospitalized but were experiencing persistent symptoms following their initial infection. The program accepted referrals from the entire province. Information regarding whether the patient was admitted to hospital or intensive care unit (ICU) was indicated by the referring practitioner.

Prior SARS-CoV-2 infection is confirmed for each patient is confirmed based on the presence of a positive nasopharyngeal polymerase chain reaction (PCR) swab and/or positive serology (if this was tested prior to vaccination). Patients were emailed a baseline questionnaire as a PDF file to complete independently prior to their first assessment. Patients had the option to either answer questions electronically or complete them on paper. The baseline questionnaire elicits information about employment status, ethnicity, date of COVID-19 symptom onset, current symptoms, and contains standardized patient-reported outcome measures.

In this cross-sectional study, we included consecutive adult patients who tested positive for COVID-19 between March 1, 2020 and July 17, 2021, and completed their baseline questionnaire at least 28 days after testing positive. These dates encompassed the first three waves of COVID-19 in BC, and during this period, there were 149,308 total cases reported in the province, of which 8117 (5.4%) were hospitalized and 1847 (1.2%) required ICU [24]. Patients were excluded if there was missing information about the date of the confirmed positive COVID-19 test or if their COVID-19 hospitalization history was not known. We analyzed the previously hospitalized and non-hospitalized patients in parallel as two independent cohorts given their different referral criteria.

### Outcome measures

The FSS is a self-administered instrument which takes about 8 min to complete [25]. It includes 9 items, each consisting of a statement for which respondents are asked to indicate their level of agreement from 1 (strongly disagree) to 7 (strongly agree) [20]. Higher scores for each item indicate greater fatigue severity. We scored the FSS by calculating the mean score of the nine items [26]. We computed a score if ≥ 8 items were completed, which is considered acceptable given that FSS items are unidimensional and strongly correlated with each other [27]. An FSS score ≥ 4 indicates clinically important fatigue [21, 28].

The EQ-5D-5L measures health-related quality of life (HRQOL) based on five items that each represent a domain (Mobility, Self Care, Usual Activities, Pain/Discomfort, and Anxiety/ Depression) [29]. Patients rate their health status on a five-point scale for each domain (no problems, slight problems, moderate problems, severe problems or extreme problems), which represents a “health state” that supports calculation of a health utility score using a value set algorithm derived from the preferences of a particular population. In this study, we derived health utilities from patient responses using a Canadian value set where the scores can range from -0.148 for the worst health state to 0.949 for the best [30]. The EQ-5D-5L also consists of a visual analogue scale (VAS) in which patients are asked to indicate their health that day from 0 (worst health imaginable) to 100 (best health imaginable) [29].

The PHQ-2 is a widely used screening instrument for depression that consists of two items that ask about depressed mood and anhedonia [31]. Patients are asked to indicate the frequency of each symptom over the past 2 weeks, from 0 (not at all) to 3 (nearly everyday). The maximum total score is 6 and a score ≥ 3 is considered a positive screen, with 92% specificity for detecting major depression [32].

The questionnaire also contained two SISQs that screened for fatigue. First, “fatigue” was listed as part of a symptom inventory in which respondents indicated with a check box whether the symptom was currently present. In a subsequent section titled “medical status”, respondents were asked to indicate “yes” vs. “no” on whether they had particular conditions or problems from a list, and one of these items was “always feeling tired”.

### Statistical analyses

The methods used to evaluate the psychometric properties of the FSS in this study have been applied previously in the context of other disease groups and healthy populations [25, 28, 33, 34, 35]. The hospitalized and non-hospitalized patient cohorts were assessed in parallel using the same methods. The analyses were conducted using Microsoft Excel and IBM SPSS.

#### Data quality and distributions

Data quality was assessed by calculating the proportion of FSS questionnaires that had missing scores and the proportion for which mean FSS scores could be computed. We examined the distribution of scores for by calculating the mean, standard deviation and skewness for individual items and overall FSS score, and by assessing for ceiling effects and floor effects. Typically, ceiling or floor effects are considered present if greater than > 15% of respondents have the minimum (FSS of 1) or maximum scores (FSS of 7) respectively [36].

#### Internal consistency

The internal consistency of the FSS was assessed by measuring the correlation between each item and between each item and the overall FSS score. The item-FSS correlation score was corrected for overlap by comparing the correlation between the item and the mean of all other items on the FSS. A separate Cronbach’s alpha statistic was calculated for each patient cohort, with additional calculations leaving out each individual FSS item. A Cronbach’s alpha of > 0.9 is considered appropriate internal consistency [37].

#### Construct validity

Construct validity is assessed by comparing the measure of interest to other constructs that are known to be positively associated (i.e. convergent validity) and to constructs that are known to be unrelated or negatively associated (i.e. divergent validity) [38, 39]. For other disease populations, the construct validity of the FSS has been assessed through its comparison with patient-reported symptoms of depression and EQ-5D scores [33, 34]. One study also demonstrated that among the EQ-5D dimensions, FSS had the strongest negative correlation with the “usual activities” dimension [40]. In this study, we hypothesized that patients who screened positive for depression on the PHQ-2 would have higher FSS scores, that there would be inverse relationships between FSS and HRQOL, and between FSS and ability to perform usual activities. Spearman correlation was used to quantify associations between FSS and EQ-5D-5L health utility and FSS and EQ-5D VAS score. The Mann–Whitney U test and Kruskal–Wallis test were used to determine the between group differences between FSS scores based on PHQ-2 and the EQ-5D-5L usual activities dimension respectively.

#### Evaluation of single item screening questions

We examined how responses to these SISQs were able to classify fatigue in comparison to the FSS based on the FSS cut-off score of ≥ 4. This was done by calculating the sensitivity, specificity, positive likelihood ratio (PLR), negative likelihood ratio (NLR), positive predictive value (PPV), and negative predictive value (NPV). Among patients who answered both SISQs on the questionnaire, we assessed the degree of agreement between these questions using the Cohen’s Kappa statistic. A Kappa statistic of 0.41–0.60 is considered moderate, 0.61–0.80 substantial, and 0.81–1.0 almost perfect agreement [41].

## Results

### Study populations

A total of 1402 consecutive patients completed questionnaires during the eligibility period. Of these, 308 were removed due to having completed the questionnaire < 28 days following their COVID-19 diagnosis or having incomplete information about COVID-19 diagnosis date, questionnaire date or hospitalization status. Of the remaining 1094 patients, 548 were previously hospitalized for acute COVID-19 and 546 were not hospitalized. The sociodemographic and clinical characteristics of these groups is outlined in Table 1. Hospitalized patients answered questionnaires at a mean of 111.0 days since diagnosis, were a mean age of 57.3, 44.2% were female and 31.6% were white. Non-hospitalized patients answered questionnaires at mean of 158.6 days since their COVID-19 diagnosis, with a mean age of 45.4 years, 67.9% female and 49.3% white.

**Table 1:** Characteristics of study population

	Hospitalized, *n* (%) or mean ± SD	Non-hospitalized, *n* (%) or mean ± SD
Total patients	548 (100)	546 (100)
Time since diagnosis (days)	111.0 ± 52.4	158.6 ± 73.3
Time since symptom onset (days)	115.3 ± 60.4	163.9 ± 85.0
Age (years)	57.3 ± 14.3	45.4 ± 12.9
Female sex	242 (44.2)	371 (67.9)
Employment status		
Unemployed	229 (41.8)	84 (15.4)
Employed, work full time	182 (33.2)	233 (42.7)
Employed, work part time	39 (7.1)	97 (17.8)
Employed but unable to work	83 (15.1)	97 (17.8)
Missing or declined to answer	15 (2.7)	35 (6.4)
Ethnicity		
Arab/ West Asian	15 (2.7)	7 (1.3)
Black	9 (1.6)	5 (0.9)
Chinese	59 (10.8)	20 (3.7)
Filipino	67 (12.2)	17 (3.1)
Indigenous	28 (5.1)	16 (2.9)
Japanese	4 (0.7)	3 (0.5)
Korean	1 (0.2)	2 (0.4)
Latin American	21 (3.8)	18 (3.3)
South Asian	101 (18.4)	118 (21.6)
Southeast Asian	17 (3.1)	7 (1.3)
White	173 (31.6)	269 (49.3)
Other	32 (5.8)	27 (4.9)
Missing or declined to answer	21 (3.8)	37 (6.8)
Intensive care unit stay	193 (35.2)	N/A

*SD* standard deviation

### Data quality and distributions

**Fig. 1:**
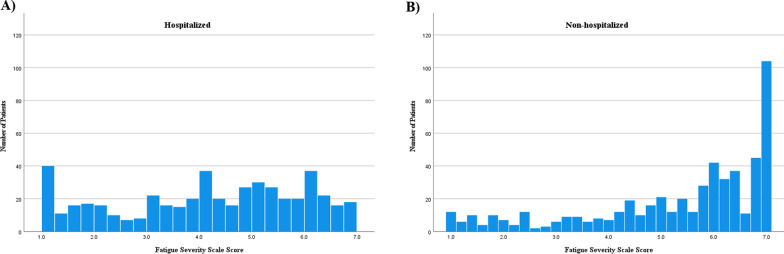
Histograms of distribution of FSS in **A** Hospitalized, and **B** Non-hospitalized patients

Among hospitalized patients, 490 (89.4%) completed all 9 items on the FSS and 13 (2.4%) returned questionnaires that had no items completed. In the non-hospitalized group, 518 (94.7%) completed all items and 4 (0.7%) no items. FSS scores were computed if at least 8 items were complete, and this comprised 536 (98.2%) hospitalized patients and 528 (96.4%) non-hospitalized patients. The distributions of each group are displayed in Fig. 1. The mean score, ceiling effects, floor effects, and skewness of the distributions of each of the individual FSS items and overall FSS score for each group are reported in Table 2. Among the hospitalized patients, the mean (SD) FSS score was 4.4 (1.8), with ceiling effects of 7.6%, ranging from 16.1% to 31.4% for individual items. In the non-hospitalized group, the mean (SD) FSS score was 5.2 (1.7) with a negative skew of − 1.0. The ceiling effects were 16.2% for the overall FSS score and ranged between 33% and 54.7% for the individual items.

**Table 2:** Characteristics of fatigue severity scale scores

FSS items	Hospitalized					Non-hospitalized				
	*n* (%)	Mean ± SD	Floor *n* (%)	Ceiling *n* (%)	Skew	*n* (%)	Mean (SD)	Floor *n* (%)	Ceiling *n* (%)	Skew
1	529 (96.5)	4.8 ± 2.1	72 (13.6)	166 (31.4)	− 0.6	541 (99.1)	5.9 ± 1.7	26 (4.8)	296 (54.7)	− 1.6
2	524 (95.8)	4.4 ± 2.0	75 (14.3)	111 (21.2)	− 0.3	536 (98.2)	5.0 ± 2.0	55 (10.3)	194 (36.2)	− 0.7
3	529 (96.5)	4.5 ± 2.1	78 (14.7)	126 (23.8)	− 0.4	537 (98.4)	5.4 ± 1.8	36 (6.7)	203 (37.8)	− 1.0
4	527 (96.2)	4.5 ± 2.1	70 (13.3)	124 (23.5)	− 0.4	540 (98.9)	5.3 ± 1.9	43 (7.9)	210 (38.5)	− 1.0
5	526 (96.0)	4.0 ± 2.1	100 (18.2)	88 (16.1)	− 0.1	536 (98.2)	5.0 ± 2.0	57 (10.6)	178 (33.2)	− 0.7
6	529 (96.5)	4.3 ± 2.1	87 (16.4)	107 (20.2)	− 0.2	536 (98.2)	5.0 ± 2.1	67 (12.5)	177 (33.0)	− 0.8
7	527 (96.2)	4.3 ± 2.1	90 (17.1)	105 (19.9)	− 0.3	538 (98.5)	5.1 ± 2.0	61 (11.3)	196 (36.4)	− 0.9
8	530 (96.7)	4.4 ± 2.2	96 (18.1)	132 (24.9)	− 0.3	539 (98.7)	5.3 ± 2.1	65 (12.1)	240 (44.5)	− 1.0
9	529 (96.5)	4.2 ± 2.2	117 (22.1)	121 (22.9)	− 0.2	539 (98.7)	5.3 ± 2.1	57 (10.6)	248 (46.0)	− 1.0
FSS Score*	528 (96.4)	4.4 ± 1.8	31 (5.9)	40 (7.6)	− 0.3	536 (98.2)	5.2 ± 1.7	12 (2.2)	87 (16.2)	− 1.0

*FSS* fatigue severity scale; *SD* standard deviation

^*^Calculated if at least 8/9 items completed

### Internal consistency

In the hospitalized and non-hospitalized patients, the Cronbach’s alpha for the FSS was 0.96 in both groups. The individual item correlation and Cronbach’s alpha analyses for the hospitalized and non-hospitalized is shown in Additional file 1: Table S1.

### Construct validity

Construct validity of the FSS was very good in both groups. FSS scores showed a moderate negative correlation to HRQOL as measured by the EQ-5D-5L (Table 3). In the hospitalized patients, spearman correlations between FSS the EQ-5D-5L VAS scores were − 0.5 (*p* < 0.001) and between the FSS and EQ-5D-5L HU scores was − 0.6 (*p* < 0.001). In the non-hospitalized group, these correlations were − 0.5 (*p* < 0.001) and − 0.6 (*p* < 0.001) respectively. The Usual Activities dimension of the EQ-5D-5L was used to compare FSS with level of functioning (Table 4). In both the hospitalized and non-hospitalized groups, the mean FSS differed between levels of functioning, generally increasing with greater dysfunction (*p* < 0.001 for both hospitalized and non-hospitalized groups). FSS was also higher among hospitalized patients who had a positive depression screen (PHQ-2 score ≥ 3). As shown in Table 4, among the hospitalized cohort, the mean FSS scores were 5.4 among those with positive screens vs. 4.0 (*p* < 0.001). Similarly, in the non-hospitalized patients, the mean FSS was 5.9 in patients who screened positive compared to 4.9 in those that did not (*p* < 0.001).

**Table 3:** Correlation of FSS Scores with health-related quality of life (HRQOL) measures

	Hospitalized		Non-hospitalized	
	Mean ± SD	Correlation with FSS (Spearman’s rho)	Mean ± SD	Correlation with FSS (Spearman’s rho)
HRQOL: EQ-5D VAS (0–100)	65.1 ± 22.8 (*n* = 548)	− 0.5* (*n* = 528)	57.1 ± 20.1 (*n* = 546)	− 0.5* (*n* = 536)
HRQOL: EQ-5D health utility (0–1.0)	0.7 ± 0.2 (*n* = 531)	− 0.6* (*n* = 516)	0.7 ± 0.2 (*n* = 533)	− 0.6* (*n* = 524)

*HRQOL* health related quality of life; *EQ-5D* EuroQol-5 dimensions; *VAS* visual analogue scale; *FSS* fatigue severity scale; *SD* standard deviation

^*^p < 0.001, two tailed

**Table 4:** FSS by EQ-5D- usual activities and PHQ-2 depression screen

	Hospitalized			Non-hospitalized		
	*N* (%)	Mean ± SD	*P*	*N* (%)	Mean ± SD	*p*
EQ-5D usual activities						
No problems	169 (32.4)	3.0 ± 1.6	< 0.001	90 (16.8%)	3.6 (1.7)	< 0.001
Slight problems	161 (30.8)	4.5 ± 1.5		124 (23.1%)	4.7 (1.6)	
Moderate problems	124 (23.8)	5.3 ± 1.4		205 (38.2%)	5.8 (1.4)	
Severe problems	46 (8.8)	6.1 ± 1.1		81 (15.1%)	6.2 (1.3)	
Unable to perform	22 (4.2)	5.9 ± 1.8		34 (6.3%)	6.4 (1.2)	
PHQ-2 depression screen						
Negative (PHQ-2 score 0–2)	394 (74.8)	4.0 ± 1.8	< 0.001	334 (63.4%)	4.9 (1.8)	< 0.001
Positive (PHQ-2 score ≥ 3)	133 (25.2)	5.4 ± 1.5		193 (36.6%)	5.9 (1.3)	

*EQ-5D* EuroQol-5 dimensions; *PHQ-2* patient health questionnaire-2; *SD* standard deviation

### Evaluation of single item screening questions

A total of 330 (62.5%) hospitalized and 423 (78.9%) non-hospitalized patients seen in clinic were classified as fatigued according to the FSS cut-off score ≥ 4 (Additional file 1: Table S2). For the hospitalized and non-hospitalized cohorts respectively, the sensitivity of the “fatigue present” SISQ for classifying fatigue was 70.6% and 83.2%, whereas the specificity was 70.2% and 57.5%. The “always feeling tired” SISQ had a sensitivity of 70.5% and 89.6% and specificity of 76.4% and 58.7%. The positive and negative likelihood ratios were also calculated (Table 5) as well as the positive and negative predictive values (Additional file 1: Table S3).

**Table 5:** Sensitivity, specificity and likelihood ratios of single item screening questions for fatigue (FSS ≥ 4)

SISQ	Hospitalized				Non-hospitalized			
	Sensitivity	Specificity	PLR	NLR	Sensitivity	Specificity	PLR	NLR
Fatigue present	70.6%	70.2%	2.4	0.4	83.2%	57.5%	2.0	0.3
Always feeling tired	70.5%	76.4%	3.0	0.4	89.6%	58.7%	2.2	0.2

*FSS* fatigue severity scale; *SISQ* single item screening question; *PLR* positive likelihood ratio; *NLR* negative likelihood ratio

Inter-item agreement between the two SISQs was also assessed for each patient group (Additional file 1: Table S4). The kappa statistic was 0.4 for both the hospitalized and non-hospitalized groups, indicating moderate agreement.

## Discussion

In this study, we performed validation assessments of the FSS instrument and two SISQs for fatigue in patients recovering from COVID-19. These assessments were completed as part of standardized evaluations within a learning health system clinical care model, and included outpatients from across BC who either were hospitalized for acute COVID-19, or were not hospitalized, but were referred for persistent symptoms. Based on current CDC and NICE criteria, the group of non-hospitalized patients would all be classified as having long COVID [42, 43]. To our knowledge, this is the largest observational study in which the FSS was used in post-hospitalization COVID-19 and/or long COVID patients.

Our findings highlight that fatigue is both common and severe in those recovering from COVID-19. To provide a context regarding the degree of fatigue severity, we can make crude comparisons between the FSS scores and those reported in the literature for healthy populations and other disease groups. For example, the fatigue severity in the hospitalized patients (mean FSS score 4.2) was over one standard deviation above the mean from what has been reported previously for healthy individuals (FSS score 3.0) [28]. The mean FSS of 5.2 in non-hospitalized patients was nearly two standard deviations above this standard, and is as high or nearly as high as what has been reported in the largest studies for conditions in which fatigue is a cardinal symptom, such as post-polio syndrome (FSS score 5.2) [44], chronic fatigue syndrome (CFS) (FSS score 6.0), fibromyalgia (FSS score 5.9) [45].

However, these relatively high FSS scores also manifested in distributions that were negatively skewed in both patient groups. This pattern was particularly pronounced in the non-hospitalized patients where we identified significant ceiling effects (16.2%), and this may represent a limitation for using the FSS in future clinical studies [36]. Specifically, it will be challenging to differentiate levels fatigue severity among patients who have maximum or near-maximum scores, and assess responsiveness to change [36]. Researchers should take this into account by considering non-parametric tests and data transformation. This negatively skewed data is also a recognized limitation of generic fatigue instruments in patients with CFS [46], but several of these instruments, including the FSS continue to be used in clinical trials [47, 48].

Although the psychometric properties of the FSS have previously been assessed in multiple patient groups [26, 33] it was important to assess this specifically in patients following COVID-19. Modern psychometric theory emphasizes that the performance of a particular survey instrument like the FSS is not a fixed property of the scale itself, but rather a function of the scale, the circumstances of administration and the specific group of respondents [38]. In this study, the FSS was acceptable in these patients as we were able to compute scores in over 96% of respondents. It was demonstrated that the FSS has strong internal consistency with high Cronbach’s alpha and inter-item correlations. Furthermore, the FSS also demonstrated construct validity. Similar to what has been reported in other health conditions, there was a moderate negative correlation with EQ-5D health utility and VAS scores [27, 40, 49]. As expected, the FSS was higher in individuals with greater impairment of their usual activities and in those who screened positive for depression [25, 27, 40].

The performance of screening questions for fatigue had not been specifically investigated in COVID-19 patients prior to this study despite widespread use during the pandemic. Two SISQs were evaluated in this study in relation to the FSS. The first SISQ used the term “fatigue”, and the second used the phrase “tired all the time”. Our analyses revealed that in the in the non-hospitalized group, both SISQ had relatively high sensitivities (> 80%) for identifying fatigue (FSS score ≥ 4), but low specificity (< 60%). In contrast, the non-hospitalized patients had both moderate sensitivities and specificities (all 60–80%). This finding is important to highlight given that several highly cited studies in the post COVID-19 literature are based on hospitalized patients and examined fatigue using similar screening questions [7, 8, 9, 10]. The higher false negative rate in this hospitalized group suggests that the prevalence of fatigue reported by these studies may in fact be underestimates. Ultimately, more comprehensive instruments such as the FSS are required to fully capture the number of patients who report fatigue.

Of the two SISQ questions, the one using the phrase “tired all the time” had slightly better performance, with sensitivity and positive likelihood ratios that were either higher or nearly identical to the SISQ that just used the term “fatigue”. It is interesting that this SISQ question was more effectively able to identify patients with fatigue as defined by the FSS despite this screening question not using the term “fatigue”. This may be an indication that longer more descriptive statements are more effective at capturing the presence of symptoms as opposed to single words (like “fatigue”, “pain”, “depression”, etc.). Our finding that there was moderate but not strong agreement between responses to these SISQs is another indication that although they are similar, these two SISQs are not always interpreted identically and researchers must consider word choice carefully when developing symptom inventories in their questionnaires.

Our study had several elements that increase its generalizability and therefore applicability to future studies. Firstly, it included multiple centres and comprised of a diverse group of patients from throughout BC that completed the FSS assessments as part of clinical care. Secondly, the stringent referral criteria ensured that the analyses were limited to patients who were referred by a clinician and were confirmed to have had COVID-19. This contrasts with research in which participation is voluntary and those in which COVID-19 status is self-reported. These other study approaches likely suffer from greater collider bias [50] and are subject to the inclusion of patients who erroneously report their COVID-19 status [51]. Lastly, this work can be applied to groups of either hospitalized or non-hospitalized patients as these were analyzed separately for this study.

However, the study had limitations that must be acknowledged. Firstly, there are both referral and non-response biases that affected the composition of the study groups. Patients that were more symptomatic were likely overrepresented as they were more likely to have been referred by their physician, and this was especially true in the non-hospitalized group. Furthermore, the inclusion criteria permitted a wide range of follow-up time points relative to initial COVID-19 illness, and this is in part a reflection of the lack of precise definition for post-COVID-19 conditions. It is also important to recognize that our analyses did not include information on the COVID-19 vaccination status. Lastly, there are some validation analyses that were not conducted, including test–retest reliability and responsiveness to change over time. Future studies should also consider the inclusion of multiple fatigue instruments and a healthy control group for comparison.

## Conclusions

In conclusion, this study demonstrated that fatigue was common and severe in patients recovering from COVID-19. The FSS is a suitable instrument to evaluate fatigue in these patients, and may be a useful tool in future studies, including clinical trials. However, investigators should be aware that ceiling effects may be a limitation in non-hospitalized patients who are referred for persistent symptoms. SISQs for fatigue can be effective, but should be used with caution as these have only modest sensitivity in some groups and may therefore provide inaccurate estimates of fatigue prevalence.

## Supplementary Information

**Additional file 1**. Supplementary tables S1–S4.

## Data Availability

The datasets generated and/or analysed during the current study are not publicly available, but are available from the corresponding author on reasonable request.

## Abbreviations

BC: British Columbia
CDC: Center for Disease Control
CFS: Chronic fatigue syndrome
EQ-5D: EuroQol 5-Dimensions
FSS: Fatigue severity scale
HRQOL: Health-related quality of life
ICU: Intensive care unit
NICE: National i\Institute for Health and Care Excellence
NLR: Negative likelihood ratio
NPV: Negative predictive value
PCR: Polymerase chain reaction
PCRC: Post COVID-19 Recovery Clinic
PDF: Portable document format
PHQ-2: Patient health questionnaire-2
PLR: Positive likelihood ratio
PPV: Positive predictive value
REB: Research ethics board
SD: Standard deviation
SISQ: Single item screening question
VAS: Visual analogue scale
