# Supplementary material for: Evaluating fatigue in patients recovering from COVID-19: validation of the fatigue severity scale and single item screening questions
Source: Health Qual Life Outcomes. 2022 Dec 27;20:170. doi: 10.1186/s12955-022-02082-x (PMC9792925; doi:10.1186/s12955-022-02082-x)
Supplement: Supplementary file 1 — Additional file 1. Supplementary tables S1–S4. [file 12955_2022_2082_MOESM1_ESM.docx]

**Supplementary Tables**

**Table S1:** Internal consistency of the FSS scores

| **FSS items** | **Hospitalized** | | | **Non-hospitalized** | | |
| --- | --- | --- | --- | --- | --- | --- |
|  | Inter-item correlations (range) | Corrected item to total correlation | Cronbach’s Alpha if item removed | Inter-item correlations | Corrected item to total correlation | Cronbach’s Alpha if item removed |
| 1 | 0.6-0.7 | 0.7 | 1.0 | 0.5-0.7 | 0.7 | 1.0 |
| 2 | 0.6-0.7 | 0.7 | 1.0 | 0.5-0.7 | 0.7 | 1.0 |
| 3 | 0.7-0.8 | 0.9 | 1.0 | 0.7-0.8 | 0.9 | 1.0 |
| 4 | 0.7-0.9 | 0.9 | 1.0 | 0.7-0.9 | 0.9 | 1.0 |
| 5 | 0.6-0.9 | 0.9 | 1.0 | 0.6-0.9 | 0.9 | 1.0 |
| 6 | 0.6-0.9 | 0.9 | 1.0 | 0.6-0.9 | 0.9 | 1.0 |
| 7 | 0.6-0.9 | 0.9 | 1.0 | 0.6-0.9 | 0.9 | 1.0 |
| 8 | 0.6-0.9 | 0.9 | 1.0 | 0.6-0.9 | 0.9 | 1.0 |
| 9 | 0.6-0.9 | 0.9 | 1.0 | 0.6-0.9 | 0.9 | 1.0 |

FSS- fatigue severity scale

**Table S2:** Responses to single item screening questions in relation to FSS scoring

|  | **Hospitalized** | | **Non-hospitalized** | |
| --- | --- | --- | --- | --- |
|  | Not fatigued (FSS <4)  (n = 113) | Fatigued (FSS ≥4)  (n = 330) | Not fatigued (FSS <4)  (n = 113) | Fatigued (FSS ≥4)  (n = 423) |
| **Fatigue present by SISQ** | | | | |
| Yes | 48 (42.5%) | 233 (70.6%) | 48 (42.5%) | 352 (83.2%) |
| No | 65 (57.5%) | 97 (29.4%) | 65 (57.5%) | 71 (16.8%) |
| Did not answer | 0 (0.0%) | 0 (0.0%) | 0 (0.0%) | 0 (0.0%) |
| **Always feeling tired by SISQ** | | | | |
| Yes | 45 (16.2%) | 232 (83.8%) | 45 (10.8%) | 372 (89.2%) |
| No | 64 (39.8%) | 97 (60.2%) | 64 (59.8%) | 43 (40.2%) |
| Did not answer | 4 (80.0%) | 1 (20.0%) | 4 (33.3%) | 8 (66.7%) |

SISQ- single item screening question; FSS- fatigue severity scale

**Table S3:** Positive and negative predictive value of single item screening questions for identifying fatigue as categorized by FSS (≥4).

| **Single item screening question** | **Hospitalized** | | **Non-hospitalized** | |
| --- | --- | --- | --- | --- |
|  | PPV | NPV | PPV | NPV |
| **Fatigue present** | 79.8% | 58.9% | 88.0 % | 47.8% |
| **Always feeling tired** | 83.8% | 60.1% | 89.2% | 59.8% |

PPV- positive predictive value; NPV- negative predictive value

**Table S4:** Agreement between single item screening questions

|  | **Hospitalized (N=539)** | | | **Non-hospitalized (N=531)** | | |
| --- | --- | --- | --- | --- | --- | --- |
|  | Fatigue not present | Fatigue present | Kappa | Fatigue not present | Fatigue present | Kappa |
| Not tired all the time | 166 | 85 | 0.4* | 68 | 40 | 0.4* |
| Tired all the time | 75 | 213 |  | 65 | 358 |  |

*p<0.001
